# Safety and Efficacy of TruBlk™ Shilajit Resin Supplementation on Physical Performance and Blood Biomarkers in Healthy Adults: A 28-Day Open-Label Pilot Study

**DOI:** 10.7759/cureus.102372

**Published:** 2026-01-27

**Authors:** Divya Yadav, Sanjay Mishra, Karan M Shah, Surya T Reddy, Rohit Gupta, Pramod Pandey

**Affiliations:** 1 Research and Development, Amaara Botanicals Private Limited, Gurugram, IND; 2 Clinical Research, Bensups Hospital, New Delhi, IND; 3 Clinical Trials and Pharmacovigilance, Auriga Research Private Limited, Gurugram, IND; 4 Quality Assurance and Regulatory Affairs, Amaara Botanicals Private Limited, Gurugram, IND; 5 Product Development and Innovation, Amaara Botanicals Private Limited, Gurugram, IND

**Keywords:** biomarkers, endurance, ergogenic aid, inflammation, mumie, muscle strength, physical performance, resin, salajit, s: shilajit

## Abstract

Background

Shilajit is a mineral-rich natural exudate traditionally used as a rejuvenator and performance enhancer. While previous studies have evaluated standardized Shilajit extracts, there is limited clinical evidence on the traditional resin formulation. This study aimed to assess the safety and efficacy profile of Shilajit resin supplementation on physical performance parameters and blood biomarkers in healthy, moderately active adults.

Methods

This was an open-label, single-arm pilot clinical study conducted over 28 days in 25 healthy male participants aged 21-55 years. Participants received 500 mg/day of TruBlk™ Shilajit Resin (250 mg twice daily). Primary outcomes included changes in muscle strength (one-repetition maximum [1RM] leg press), muscle endurance, grip strength, fatigue severity (Fatigue Severity Scale [FSS]), and rating of perceived exertion (RPE). Secondary outcomes included aerobic capacity (maximal oxygen uptake [VO₂ max]), body composition parameters, inflammatory markers like C-reactive protein (CRP) levels, muscle damage markers such as creatine kinase (CK) and lactate dehydrogenase (LDH), and hormonal markers (total testosterone). Safety was evaluated through adverse event monitoring and laboratory assessments, including liver and kidney function tests and hematological parameters.

Results

After 28 days of supplementation, statistically significant and large magnitude within-group improvements were observed in 1RM leg press strength (+12.94%, p < 0.001), muscle endurance (+12.30%, p < 0.001), grip strength of the dominant hand (+5.73%, p = 0.038), and VO₂ max (+1.36%, p < 0.001). FSS scores decreased by 32.40% (p < 0.001), and RPE scores decreased by 23.63% (p < 0.001). CRP levels significantly declined by 25.35% (p = 0.023). Further, CK levels decreased by 41.70% (p = 0.059, a non-significant trend that, considering the high inter-individual variability at baseline, suggests a potential meaningful reduction in exercise-induced muscle damage and improved recovery status. This should be a key focus of future larger-scale studies. Body composition analysis demonstrated a statistically significant increase in lean body mass (+1.5%, p = 0.002) and a reduction in body fat percentage (−2.34%, p = 0.033). No serious adverse events were reported, and all safety laboratory parameters remained within normal limits.

Conclusions

Shilajit resin supplementation at a dose of 500 mg/day for 28 days was associated with statistically significant improvements in muscle strength, endurance, aerobic capacity, and fatigue parameters, along with favorable changes in inflammatory markers, while demonstrating good safety and tolerability in healthy adult males. These findings support further investigation of Shilajit resin in larger, randomized, placebo-controlled clinical trials.

## Introduction

Shilajit (also known as salajit or mumie) is a blackish-brown exudate traditionally derived from rocks in the Himalayan mountain ranges, formed over centuries through the decomposition of plant matter and microbial metabolites [1]. This substance has been used for millennia in Ayurvedic and traditional medicine systems as a rasayana (rejuvenator) to enhance physical performance, improve vitality, and promote longevity [2]. The bioactive composition of Shilajit includes fulvic acid (15-20%), humic acid, dibenzo-alpha-pyrones (DBPs), and over 80 minerals in their ionic form [3].

Recent scientific investigations have begun to validate traditional claims regarding Shilajit’s health benefits. Keller et al. (2019) demonstrated that 500 mg/day of purified Shilajit extract for eight weeks reduced fatigue-induced decline in maximal voluntary isometric contraction and lowered serum hydroxyproline in recreationally active men [4]. Pandit et al. (2016) reported significant increases in total and free testosterone, as well as dehydroepiandrosterone sulfate (DHEAS), following 90 days of supplementation with 250 mg twice daily in middle-aged men [5].

Studies examining blood biomarkers have shown promising results. Niranjan et al. (2016) found that Shilajit supplementation (250 mg twice daily for 12 weeks) improved endothelial function and reduced high-sensitivity C-reactive protein (hsCRP) and malondialdehyde (MDA) while increasing nitric oxide (NO) and glutathione (GSH) in patients with type 2 diabetes [6]. Similarly, Patil et al. (2023) observed decreased MDA and oxidized low-density lipoprotein (ox-LDL) alongside increased total antioxidant capacity, superoxide dismutase (SOD), and reduced GSH in elderly hypertensive patients receiving 500 mg twice daily for 30 days [7].

Mechanistic studies suggest that Shilajit’s effects are mediated through enhanced mitochondrial bioenergetics, antioxidant activity, and anti-inflammatory pathways. Das et al. (2016) demonstrated that Shilajit supplementation upregulated genes involved in extracellular matrix remodeling, mechanotransduction, and muscle repair without increasing creatine kinase (CK) or myoglobin [8]. 

Despite these promising findings, significant gaps preclude a clear understanding of Shilajit’s role as an ergogenic aid. Critically, nearly all prior human trials have investigated purified extracts, not the traditional resin formulation purported to contain the full bioactive spectrum. Furthermore, research has focused on clinical populations or isolated biomarkers, with a paucity of comprehensive data on functional performance outcomes (strength, endurance, and power) in the healthy, active demographic most likely to use it for performance enhancement [9]. The resin form represents the most natural and potentially bioactive preparation, containing the full spectrum of compounds in their native matrix. Second, few studies have simultaneously evaluated functional performance outcomes alongside mechanistic blood biomarkers in a comprehensive assessment [9]. Third, most trials have been conducted in specific populations (middle-aged men, diabetic patients, and elderly individuals), with limited data in healthy, active adults, the primary demographic interested in performance enhancement.

This pilot study aimed to address these gaps by conducting a clinical evaluation of Shilajit resin formulation in healthy, moderately active adults. Our primary objectives were to (1) assess the effects of 28 days of Shilajit resin supplementation on muscle strength, cardiovascular endurance, and body composition; (2) evaluate changes in blood biomarkers related to inflammation, muscle damage, and hormonal status; and (3) establish the safety and tolerability profile of the resin formulation. We hypothesized that 28 days of supplementation with the traditional Shilajit resin formulation would be safe and well-tolerated and lead to measurable, favorable changes in physical performance parameters and associated blood biomarkers in healthy adults.

## Materials and methods

This single-center, open-label, single-arm, pre-post interventional pilot study was conducted from August to September 2025. The study was approved by the GSER Independent Ethics Committee under approval number GSER/2025/BMR-AP/181. The exploratory design was selected to generate preliminary efficacy and safety data prior to a definitive randomized controlled trial. The study was registered with the Clinical Trials Registry-India (CTRI/2025/08/092294) prior to participant enrollment. All study procedures adhered to the Declaration of Helsinki (2013), International Council for Harmonization Good Clinical Practice (ICH-GCP) E6(R2), and applicable Indian regulatory guidelines.

**Figure 1:**
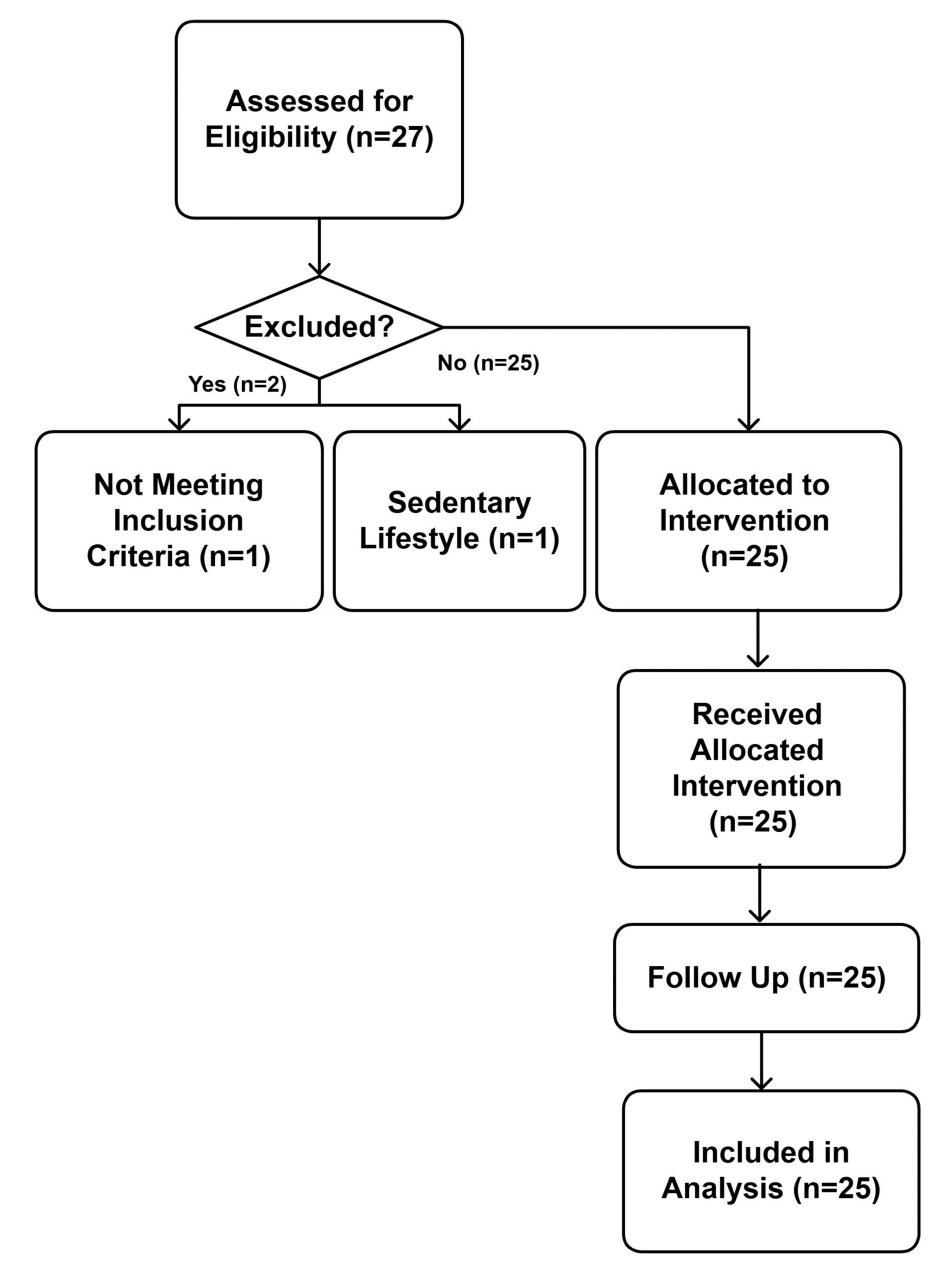
CONSORT flow diagram of participant enrollment and progress through the study n, total number of participants; CONSORT, Consolidated Standards of Reporting Trials

Healthy male adults aged 21-55 years, with a BMI of <30 kg/m² and engaging in 150-300 min of moderate-intensity physical activity per week, were eligible for participation. Exclusion criteria included metabolic, cardiovascular, renal, or hepatic disorders; recent musculoskeletal injury or surgery; use of anabolic agents, corticosteroids, or anti-inflammatory medications; known allergy to Shilajit; or participation in another trial in the preceding 30 days. Among 27 screened individuals, 25 met eligibility criteria, were enrolled, and completed the 28-day intervention period without dropouts or major protocol deviations (Figure 1).

Participants received TruBlk™ Shilajit Resin (Amaara Botanicals Private Limited, Gurugram, India) at a dose of 250 mg twice daily, dissolved in approximately 200 mL of warm water and taken with meals. The Shilajit resin used in this study was manufactured in a good manufacturing practice (GMP) facility using solvent-free aqueous extraction, standardized to ≥60% fulvic acids, confirmed for DBP markers, and tested for heavy metals, microbial load, pesticides, aflatoxins, and polycyclic aromatic hydrocarbons (PAHs) within accepted safety limits. Participant compliance was monitored through daily intake logs and verification of returned product packaging. 

Primary outcomes included muscle strength (one-repetition maximum [1RM] leg press), muscle endurance (repetitions at 70% 1RM), grip strength (digital dynamometer), Fatigue Severity Scale (FSS) score, and Borg rating of perceived exertion (RPE). Secondary outcomes included cardiovascular endurance via the modified Harvard step test, body composition using an 8-electrode bioimpedance analyzer, and fasting blood biomarkers (CRP, CK, lactate dehydrogenase [LDH], testosterone, complete blood count [CBC], and liver and kidney function markers). Vital signs and adverse events were recorded at baseline, day 14, and day 28.

Statistical analyses were performed using IBM SPSS Statistics, version 27.0 (IBM Corp., Armonk, NY). Descriptive statistics summarized continuous and categorical variables. Within-group comparisons were analyzed using paired t-tests. Test statistics (t-values with degrees of freedom), two-tailed p-values, percentage change from baseline, effect sizes (Cohen’s d), and 95% confidence intervals for mean differences were reported where applicable. A p-value of <0.05 was considered statistically significant. Given the pilot nature, no adjustments for multiple comparisons were made.

## Results

All 25 enrolled participants completed the 28-day intervention without dropouts or protocol deviations. The cohort consisted of healthy adult males with a mean age of 26.32 ± 4.29 years, a height of 174.68 ± 7.97 cm, a weight of 81.04 ± 12.10 kg, and a BMI of 26.48 ± 2.79 kg/m². Baseline vital signs were within normal physiological limits. Compliance with the supplementation regimen was high, with a mean adherence rate of 94%. Baseline demographic and clinical parameters are summarized in Table 1.

**Table 1:** Baseline demographic and clinical characteristics (N = 25) BMI, body mass index; BP, blood pressure; bpm, beats per minute

Parameter	Mean (SD)	Range
Age (years)	26.32 (4.29)	21-37
Height (cm)	174.68 (7.97)	161-196
Body weight (kg)	81.04 (12.10)	55.80-115.00
BMI (kg/m²)	26.48 (2.79)	18.9-29.9
Systolic BP (mmHg)	121.60 (6.88)	108-134
Diastolic BP (mmHg)	80.08 (6.15)	70-90
Pulse rate (bpm)	75.44 (6.07)	64-84

Primary outcomes

**Figure 2:**
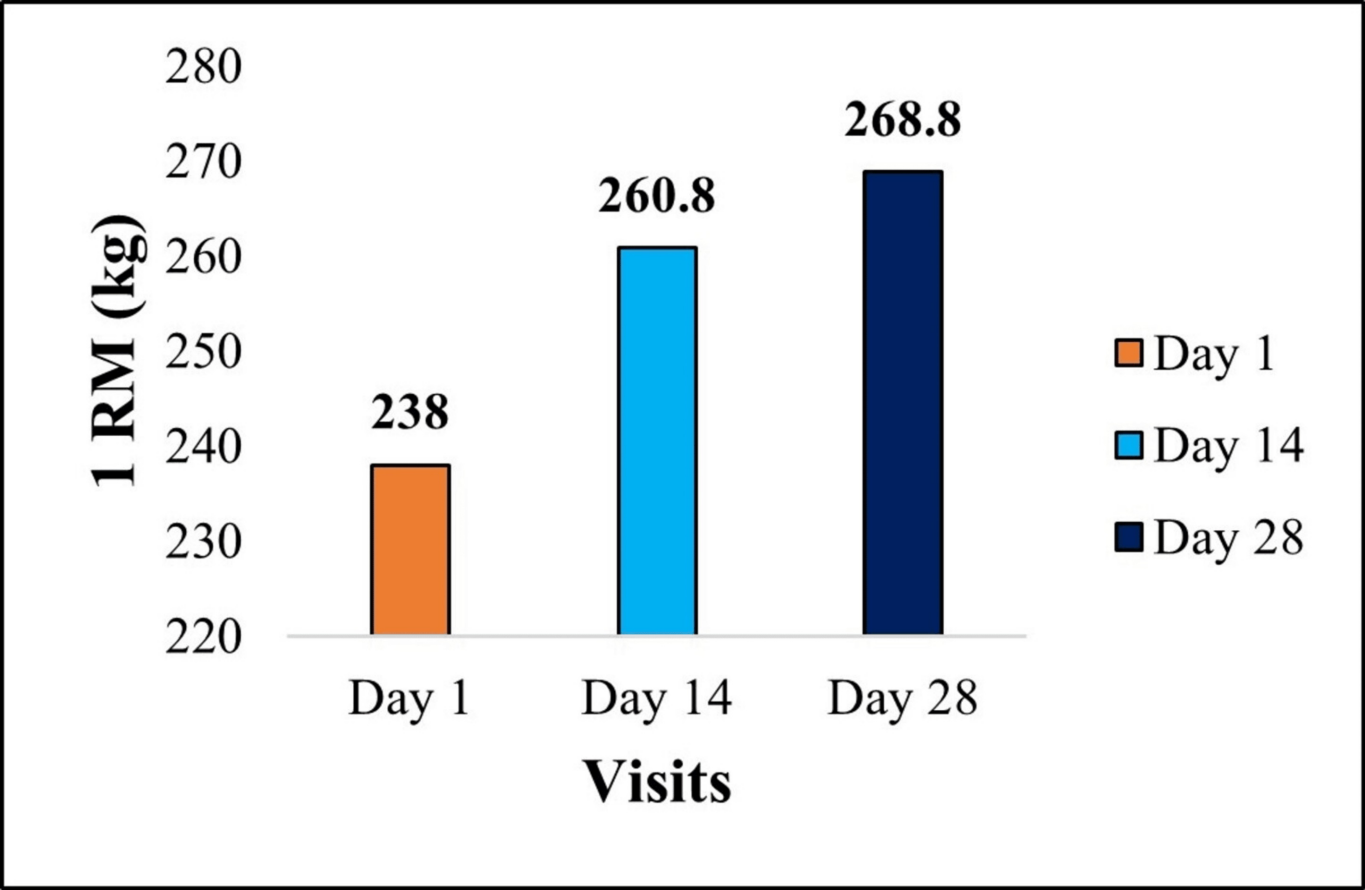
Graphical representation of muscle strength: 1RM leg press Change in 1RM leg press strength (kg) from baseline to day 28 following supplementation. Data are presented as mean. Percentage change: +12.94% improvement from baseline. Statistical significance between day 1 vs day 28: p < 0.001. Effect size (Cohen’s d): 0.96 (large), indicating a substantial improvement in muscle strength. 1RM, one-repetition maximum

Significant improvements were observed in muscle strength and endurance following 28 days of supplementation. 1RM leg press was assessed using standard procedures [10]. 1RM leg press increased from 238.0 ± 48.65 kg at baseline to 268.8 ± 49.86 kg (+12.94%). This improvement was statistically significant (paired t-test, t(24) = 4.81, p < 0.001; 95% CI: 17.59 to 44.01; Cohen’s d = 0.96), as presented in Figure 2.

**Figure 3:**
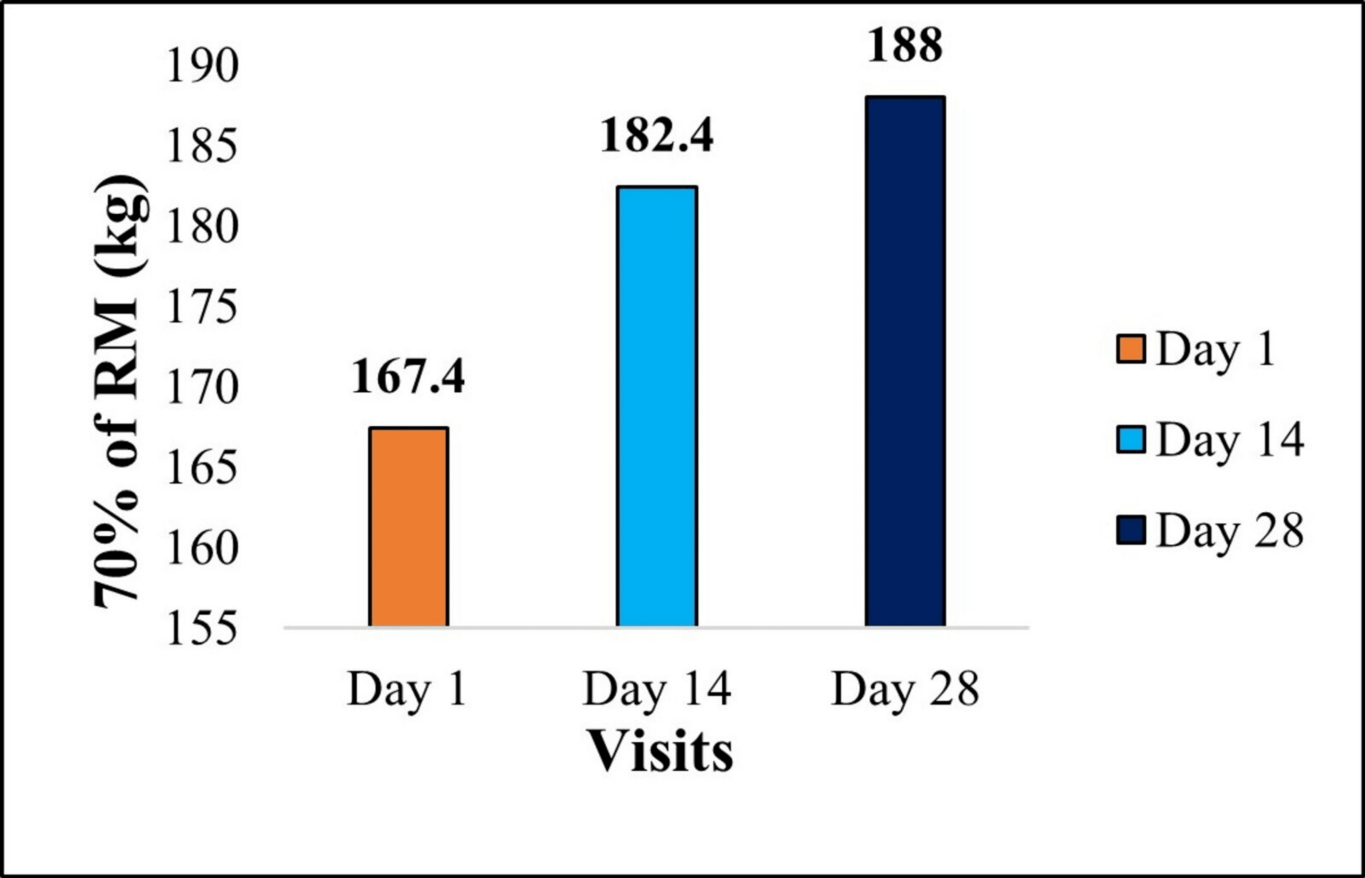
Graphical representation of changes in 70% of 1RM leg press Changes in muscle endurance, measured as the number of repetitions performed at 70% of 1RM, from baseline to day 28. Data are presented as mean. Percentage change: +12.30% improvement. Statistical Significance between day 1 vs day 28: p < 0.001. Effect size (Cohen’s d): 0.92 (large), indicating a substantial enhancement in muscular endurance. 1RM, one-repetition maximum

Muscle endurance, measured as repetitions to failure at 70% of 1RM, improved from 167.40 ± 35.15 to 188.00 ± 36.51 (+12.30%). The change was statistically significant (paired t-test, t(24) = 4.58, p < 0.001; 95% CI: 11.33 to 29.87), as presented in Figure 3.

Dominant handgrip strength increased significantly (+5.73%, paired t-test, t(24) = 2.20, p = 0.038; 95% CI: 0.15 to 4.81), whereas non-dominant hand strength showed a non-significant increase. Detailed outcomes for hand grip strength for both the dominant and non-dominant are represented in Table 2.

**Table 2:** Changes in dominant and non-dominant hand grip strength from baseline to day 28 Values are presented as mean (SD). Dominant hand grip strength showed a significant improvement from baseline to day 28, while non-dominant hand strength showed a non-significant increase. A p-value of <0.05 was considered statistically significant.

Parameter	Baseline	Day 28	Change	% Change	p-value	Effect size (Cohen’s d)
Grip strength: dominant (kg)	43.28 (7.74)	45.76 (8.33)	+2.48 (5.65)	5.73%	0.038	0.44 (medium)
Grip strength: non-dominant (kg)	40.33 (7.98)	41.93 (7.98)	1.59 (5.59)	3.94%	0.166	0.28 (small)

Secondary outcomes

FSS scores decreased progressively, with a 32.40% reduction by day 28 (paired t-test, t(24) = −7.20, p < 0.001; 95% CI: −9.57 to −5.31) [11]. RPE decreased significantly from baseline to both days 14 and 28 (−23.64%, paired t-test, t(24) = −7.77, p < 0.001; 95% CI: −1.97 to −1.15) [12]. Cardiovascular endurance demonstrated modest but significant improvement; estimated VO₂ max increased by 1.36% (paired t-test, t(24) = 4.13, p < 0.001; 95% CI: 0.31 to 0.94), while recovery heart rates at 1-, 2-, and 3-min post-exercise decreased significantly [13]. Fitness index increased by 5.86% (paired t-test, t(24) = 4.16, p < 0.001; 95% CI: 1.58 to 4.70). Body composition analysis revealed increased lean body mass (+1.51%, paired t-test, t(24) = 3.41, p = 0.002; 95% CI: 0.33 to 1.32), increased fat-free mass (+1.54%, paired t-test, t(24) = 3.47, p = 0.002; 95% CI: 0.36 to 1.43), and reduced body fat percentage (−2.34%, paired t-test, t(24) = −2.27, p = 0.033; 95% CI: −1.24 to −0.06), with minimal change in total body weight. Recovery markers showed a significant reduction in DOMS by day 28 (paired t-test, t(24) = −2.43, p = 0.023; 95% CI: −1.41 to −0.12). Secondary outcome data are summarized in Table 3.

**Table 3:** Changes in fatigue, performance, cardiovascular fitness, and body composition parameters from baseline to day 28 Values are mean (SD). Significant improvements were observed across fatigue, exertion, cardiovascular fitness, and body composition parameters following 28 days of supplementation. Paired comparisons were performed between baseline and day 28 (a p-value of <0.05 is considered significant).

Parameter	Baseline	Day 28	Change	% Change	p-value
Fatigue Severity Scale	22.96 (6.80)	15.52 (4.34)	-7.44 (5.16)	-32.40%	<0.001
Borg RPE	6.60 (1.47)	5.04 (1.31)	-1.56 (1.00)	-23.63%	<0.001
VO_2_ max (mL/kg/min)	45.71 (1.57)	46.34 (1.30)	0.62 (0.75)	+1.36%	<0.001
Recovery heart rate 1 min (bpm)	102.00 (13.33)	94.08 (10.16)	-7.92 (7.15)	-7.76%	<0.001
Fitness index	53.57 (7.83)	56.72 (6.49)	3.14 (3.78)	+5.86%	<0.001
Body weight (kg)	81.10 (12.29)	81.58 (12.38)	0.47 (1.09)	+0.57%	0.040
Body fat (%)	27.69 (6.24)	27.04 (6.33)	-0.65 (1.43)	-2.34%	0.033
Lean body mass (kg)	54.40 (7.46)	55.23 (7.65)	0.82 (1.20)	+1.50%	0.002
Fat-free mass (kg)	58.32 (8.00)	59.22 (8.20)	0.90 (1.29)	+1.54%	0.002
Skeletal muscle mass (kg)	33.38 (4.60)	33.55 (5.01)	0.18 (2.10)	+0.53%	0.680

Blood biomarkers

CRP decreased significantly (−25.35%, paired t-test, t(24) = −2.43, p = 0.023; 95% CI: −1.35 to −0.11). CK levels decreased numerically; however, this change did not reach statistical significance (−41.70%, paired t-test, t(24) = −1.98, p = 0.059). LDH and total testosterone levels did not show statistically significant changes over the study period. These findings are summarized in Table 4.

**Table 4:** Changes in blood biomarkers Values are presented as mean (SD). C-reactive protein showed a significant reduction after 28 days, while changes in muscle damage and hormonal markers were not statistically significant. Paired comparisons were conducted between baseline and day 28, with a p-value of <0.05 considered statistically significant.

Biomarker	Baseline	Day 28	Change	% Change	p-value
Inflammatory markers					
C-reactive protein (mg/L)	2.88 (1.69)	2.15 (2.00)	-0.73 (1.50)	-25.35%	0.023
Muscle damage markers					
Creatine kinase (U/L)	496.36 (568.95)	289.36 (182.22)	-207.00 (522.44)	-41.70%	0.059
Lactate dehydrogenase (U/L)	261.43 (48.31)	264.03 (33.75)	2.60 (53.72)	0.0099	0.811
Hormonal markers					
Total testosterone (ng/dL)	394.06 (152.94)	383.25 (150.47)	-10.81 (103.30)	-2.74%	0.606

Safety and adverse events

Safety laboratory parameters remained stable throughout the study (Table 5). Seven participants (28%) reported mild, self-limiting adverse events, with no serious events or study discontinuations (Table 6). Vital signs remained within normal limits at all assessments.

**Table 5:** Safety laboratory parameters Values are presented as mean (SD). All liver, kidney, and hematological safety parameters remained stable from baseline to day 28, indicating no adverse effects associated with the intervention. ALT, alanine aminotransferase; AST, aspartate aminotransferase; BUN, blood urea nitrogen; WBC, white blood cells; NS, not significant

Parameter	Baseline	Day 28	Change
Liver function tests			
ALT (U/L)	42.44 (18.58)	36.84 (11.57)	Stable
AST (U/L)	61.80 (37.59)	56.52 (32.53)	Stable
Total bilirubin (mg/dL)	0.68 (0.33)	0.64 (0.38)	Stable
Albumin (g/dL)	4.92 (0.17)	4.88 (0.17)	Stable
Kidney function tests			
Creatinine (mg/dL)	1.00 (0.12)	0.97 (0.15)	Stable
BUN (mg/dL)	13.85 (3.15)	11.78 (3.62)	Stable
Hematology			
Hemoglobin (g/dL)	15.29 (1.01)	15.41 (1.19)	Stable
WBC (×10³/µL)	8273.60 (2315.98)	9460.40 (2996.03)	Stable
Platelets (×10³/µL)	2.86 (0.71)	2.83 (0.74)	Stable

**Table 6:** Summary of adverse events reported during the 28-day study period All adverse events were mild in severity and resolved without medical intervention. Most events were assessed as unlikely or possibly related to the study product. No serious adverse events were reported. No participants discontinued the study due to adverse events. No clinically significant changes in vital signs were observed throughout the study period.

Adverse event	Number of participants (%)	Severity	Relationship to study product	Resolution
Acne	2 (8%)	Mild	Unlikely related	Resolved spontaneously
Rash	1 (4%)	Mild	Possibly related	Resolved spontaneously
Pruritus	1 (4%)	Mild	Unlikely related	Resolved spontaneously
Abdominal pain (upper)	1 (4%)	Mild	Unlikely related	Resolved within 2-3 days
Dyspepsia	1 (4%)	Mild	Unlikely related	Resolved spontaneously
Cough	1 (4%)	Mild	Possibly related	Resolved within 3-5 days
Pyrexia	1 (4%)	Mild	Possibly related	Resolved spontaneously

## Discussion

This pilot study is the first to clinically evaluate the effects of Shilajit in its traditional resin formulation in healthy adults. Our findings indicate that 28 days of supplementation with Shilajit resin (500 mg/day) was associated with statistically significant within-group changes across multiple domains of physical performance, including muscle strength, muscle endurance, cardiovascular fitness, and perceived fatigue and exertion. These within-group changes occurred alongside favorable changes in inflammatory and muscle damage biomarkers, while demonstrating a good safety and tolerability profile.

An increase of 12.94% in muscle strength (1RM leg press) observed in the present study suggests an improvement in baseline maximal strength. This differs from the finding of Keller et al. (2019), who reported preservation of isometric strength following a fatiguing protocol [4]. The disparity may relate to differences in outcome measures (dynamic vs isometric, rested vs post-fatigue) and highlights the need for standardized testing batteries in future research [4]. Our study extends these observations by showing meaningful within-group improvements in not only muscle strength but also muscle endurance, which increased by 12.3%, representing a functional outcome that has been less frequently reported in Shilajit research.

Cardiovascular endurance, measured via VO₂ max estimation, improved by 1.36%, which may be physiologically meaningful within a four-week timeframe for untrained and lightly trained individuals. To date, few studies have explored Shilajit’s impact on aerobic capacity, making this an important preliminary addition to the literature. The improvement may be related to reduced oxidative stress, enhanced NO production, and improved endothelial function previously reported with Shilajit supplementation [6,7].

A notable observation in our study was the 25.35% reduction in CRP, indicating a decrease in systemic inflammation. This aligns with findings from Niranjan et al. [6] and Patil et al. [7], who also observed reductions in inflammatory biomarkers in clinical populations. The present findings extend these observations to healthy, active adults, suggesting that Shilajit’s anti-inflammatory effects may also support recovery and performance enhancement in non-clinical populations. Further, CK levels decreased numerically by 41.7%; although this change did not reach statistical significance, the magnitude and direction of change are consistent with a potential reduction in exercise-induced muscle damage and improved recovery status. These observations should be interpreted cautiously and within the context of the exploratory study design. Nonetheless, the findings are directionally consistent with the transcriptomic results reported by Das et al. [8], who demonstrated upregulation of genes associated with muscle repair pathways following Shilajit supplementation.

Additionally, we observed a significant reduction in fatigue (−32.4%) and perceived exertion (−23.6%), suggesting improvements in both physiological and psychological responses to exercise. These findings align with previous work demonstrating Shilajit’s anti-fatigue effects, including reduced behavioral fatigue in animal models and maintenance of muscular function under stress [13,14]. 

A distinctive aspect of this study is the evaluation of Shilajit in its traditional resin form, as opposed to the purified extracts used in most prior clinical research. Theoretically, the resin’s complex phytomineral matrix may offer a different bioactive profile; however, comparative trials versus standardized extracts are required to determine whether this translates into differential clinical effects. Resin may contain a broader spectrum of naturally occurring bioactive compounds, including heat-sensitive molecules and trace minerals often lost during extraction. Traditional resin also has a lipophilic matrix that may enhance the absorption of fat-soluble components. This comprehensive phytomineral profile could contribute to the pronounced positive within-group changes in both performance and recovery observed in our study. While direct comparisons with standardized extracts are not yet available, the magnitude of physiological benefits suggests that the resin formulation warrants further investigation, potentially offering superior synergistic effects.

Despite the promising results, the study has important limitations. The open-label, single-arm design without a placebo control limits causal interpretation, although the magnitude of improvement in muscle strength and reduction in CRP may exceed what would be expected from typical training adaptations alone. The modest sample size (n = 25) of healthy males aged 21-55 years restricts generalizability, and the 28-day duration does not permit assessment of long-term efficacy or safety. To address these gaps, a randomized, double-blind, placebo-controlled trial is warranted to validate efficacy and safety over long-term supplementation in a larger cohort.

Overall, this study provides the first evidence that traditional Shilajit resin may serve as a natural ergogenic aid with both performance-enhancing and recovery-enhancing properties. The convergence of objective improvements (strength, endurance, VO₂ max, and body composition) and subjective benefits (fatigue and exertion), coupled with favorable biomarker responses (reduced inflammation and muscle damage), supports potential multi-mechanistic actions involving mitochondrial bioenergetics, inflammation modulation, and enhanced muscle repair pathways.

Future research should include randomized, double-blind, placebo-controlled studies with larger and more diverse populations, longer intervention periods, exploration of dose-response relationships, and mechanistic investigations using mitochondrial assays, oxidative stress markers, and muscle imaging techniques.

## Conclusions

This pilot study demonstrates that 28 days of Shilajit resin supplementation (250 mg twice daily) produced meaningful improvements in muscle strength, muscular endurance, performance capacity, recovery markers, and body composition in healthy adult males, while maintaining an excellent safety and tolerability profile. The consistent positive responses across several physiological and functional domains are consistent with Shilajit’s traditional use and align with prior evidence supporting its safety and ergogenic potential. These findings also complement existing knowledge on physiological adaptations to training stimuli, including improvements in muscle function and fiber-type performance characteristics, suggesting that Shilajit may support or augment exercise-related adaptations in active individuals.

Although these findings are promising, they remain preliminary and should be interpreted within the limitations of a pilot study. Larger, rigorously designed randomized controlled trials are needed to validate the observed benefits, determine optimal dosing strategies, and elucidate the biological mechanisms underlying Shilajit’s effects. Such research will help establish a stronger evidence base and clarify the role of the test product Shilajit in supporting enhanced physical performance, recovery, and overall functional capacity.
